# Quantitative analysis of some important metals and metalloids in tobacco products by inductively coupled plasma-mass spectrometry (ICP-MS)

**DOI:** 10.1186/1752-153X-6-56

**Published:** 2012-06-18

**Authors:** Syed Ghulam Musharraf, Muhammad Shoaib, Amna Jabbar Siddiqui, Muhammad Najam-ul-Haq, Aftab Ahmed

**Affiliations:** 1 H.E.J. Research Institute of Chemistry, International Center for Chemical and Biological Sciences, University of Karachi, Karachi 75270, Pakistan; 2 Department of Chemistry, Bahauddin Zakariya University (B.Z.U.), Multan 60800, Pakistan; 3 College of Pharmacy, University of Rhode Island, Kingston, RI 02881, USA

**Keywords:** Metals, Metalloids, Tobacco products, ICP-MS

## Abstract

**Background:**

Large scale usage of tobacco causes a lot of health troubles in human. Various formulations of tobacco are extensively used by the people particularly in developing world. Besides several toxic tobacco constituents some metals and metalloids are also believed to pose health risks. This paper describes inductively coupled plasma-mass spectrometric (ICP-MS) quantification of some important metals and metalloids in various brands of smoked, sniffed, dipped and chewed tobacco products.

**Results:**

A microwave-assisted digestion method was used for sample preparation. The method was validated by analyzing a certified reference material. Percentage relative standard deviation (% R.S.D.) between recovered and certified values was < 5.8. Linearity value for calibration curve of each metal was 1 > r > 0.999. Improved limits of detection (LODs) were in range of ng/L for all elements. Fe, Al and Mn were found to be in the highest concentration in all types of tobacco products, while Zn, Cu, Ni and Cr were below the average concentration of 40 μg/g, and Pb, Co, As, Se and Cd were below 5 μg/g. All elements, apart from Pb, were high in concentration in dipping tobacco in comparison to other tobacco products. Generally, the order of all elemental concentration can be expressed in different tobacco products as chewing < smoked < sniffing < dipping. However, smoked and sniffing will interchange their position in the case of Mn, Cu, Se and Cd. Multivariate statistical analyses were also performed to evaluate the correlation and variations among tobacco products.

**Conclusions:**

The present study highlights the quantification of some important metals and metalloids in a wide spectrum of tobacco formulations. The outcome of this study would be beneficial for health authorities and individuals.

## Background

Tobacco use, especially in Asian countries has a long history. Five million deaths per annum, globally, are attributed to tobacco use. If this tendency continues, tobacco related mortalities will reach 8 million worldwide annually by the year 2030 [1]. Leaves of tobacco plants are used to prepare different products including smoked (cigarettes, beedi, tobacco leaves) and smokeless (sniffing, dipping, chewing) tobacco products. The composition of tobacco is multifarious. The type and number of chemical constituents varies in its different formulations. Thousands of different chemicals have been detected in tobacco smoke; 60–70 of them are proven carcinogens [2]. The only chemical which causes addiction in tobacco products is nicotine [3]; however the tobacco plant is well known to absorb trace elements from the soil and to accumulate them in its leaves on large scale. Some of these elements are toxic to human even in minute quantities [4-7]. Quantity of these trace elements in tobacco depends upon genotype, type of water, soil and their respective pH, stalk position, fertilizers, pesticides applied and the environment.

Biochemical effects of toxic and trace elements in tobacco and tobacco smoke are well documented by Chiba [8]. Among the metals, aluminum is the major ingredient in tobacco formulations. Aluminum toxicity is associated with alteration of calcium metabolism in the brain [9]. Chromium is carcinogenic in its hexavalent form. Maternal smoking has been linked to adverse effects on selenium metabolism in the developing foetus. Such women have low polymorphonuclear cell zinc concentrations, ultimately face a threat of delivering small-for-gestational-age babies [8]. The International Agency for Research on Cancer (IARC) has recently defined nickel as a Group 1 “carcinogenic to humans” [10]. Copper poisoning in humans, particularly by chewing the tobacco formulation called gutka, is a major source of fibrosis in mouth cavities [11]. Lead is more hazardous for the young ones, as its chronic exposure plays role in the lowering of intelligence quotient (IQ) levels and it is also associated with impaired foetal development [12]. Arsenic toxicity causes skin cancer, mouth ulcerations, low haemoglobin, leukaemia, acute renal failure and nerve damages [13]. High cadmium level is related to cardiovascular diseases [14].

Pakistan produces more than 75 million kilograms of tobacco annually. After meeting the domestic requirement of 45–50 million kilograms, the rest of the tobacco is exported. Tobacco utilization is constantly increasing in Pakistan, India, Bangladesh and other countries of South Asia. In Pakistan, there are 22 million smokers, according to a health survey by Pakistan Medical Research Council in 2003, 36% of adult men and 9% of adult women are smokers in Pakistan [15] and about 100,000 people die in the country each year from the diseases caused by tobacco use. Tobacco formulations such as niswar and gutka are popular among the people of South Asia and responsible for oral cancer and its related diseases. Niswar, a sniffing and dipping tobacco product, is mainly composed of tobacco leaves, calcium oxide, and wood ash while Gutka, a chewing tobacco product, is composed of crushed areca nut, tobacco, catechu, paraffin, lime and sweet syrup. Gutka is very popular among teenagers, whilst niswar is particularly used in the Pushtoon and Afghan communities in Pakistan. These tobacco product usages are increasing at an alarming rate. Moreover sniffing, dipping and chewing products may cause metal toxicity as these products are directly consumed by the users and metals can absorb directly through the mucosal membrane. Therefore, there is an important need to quantify the metals, particularly in sniffing, dipping and chewing tobacco products.

Many methods have been employed for the determination of metals in tobacco products, mainly in cigarettes [16-25]. The aim of the present work was to develop a sensitive method for the quantitative estimation of a wide range of metals and two important metalloids (As & Se) in a variety of tobacco products and to compare levels. Twelve elements, in a broad collection of Pakistani cigarette brands, niswar, sniffing niswar, and gutka formulations, were analyzed by ICP-MS using microwave digestion. International cigarettes and niswar formulations were also analyzed for a comparison of metal and metalloid’s toxicity of local and imported tobacco products. Moreover, the present study will be helpful to pick dietary intake values of these metals and metalloids, so that one can assess the risk of overdosing of these metals and metalloids. It is the first report for the determination of metals and metalloids in a wide range of tobacco products and it will be useful for health and environment authorities in Pakistan and other countries of South Asia.

## Experimental

### Chemicals and instrumentation

Nitric acid was of trace metal grade and was purchased from Thermo-Fisher, USA. ICP-MS verification standard (Tune-A) and multi-element calibration standards were purchased from Ultra Scientific, USA. Deionized water from Milli-Q system (Millipore, Bedford, MA, USA) was used exclusively. Samples were digested using the Microwave Accelerated Reaction System and associated Teflon microwave vessels (CEM Corp, Matthews, USA, X7-Series). Elemental analysis was performed using Thermo Elemental X7 Series ICP-MS System coupled with Cetac ASX-510HS high speed auto sampler (Omaha, Nebraska 68144 USA). Argon gas cylinders were connected through a six cylinder gas manifold from Western Innovator (Ohio, USA). Samples were centrifuged by Eppendorf micro-centrifuge (Hamburg, Germany).

### Sampling

Fifty five different brands of tobacco smoked, sniffing, dipping and chewing products, commercially available in Pakistan, were purchased from various markets of Karachi. Each product was purchased twice, having different batch numbers and packing dates from different shops and stored in plastic bags separately. Envelope, paper and filters were carefully removed from the cigarettes and weighed amounts of duplicate samples were pooled and crushed in liquid nitrogen with pestle and mortar. Fine powdered samples were air-dried and stored at – 4 °C in polythene bags until use. Sniffing, dipping and chewing samples were processed similarly and stored. All samples were coded and their decoding details are available in the Additional file 1.

### Microwave-assisted digestion

Samples (10–30 mg) were weighted directly into micro-vessels and 1 mL of nitric acid was added. After five minutes, vials were capped using a torque wrench of capacity 20 in-lb (≅ 2.26 Nm) with a 22 mm socket. 10 mL of water was added into each HP500 plus standard digestion vessel. Two Teflon reaction vessels were inserted carefully in each digestion vessel. The micro-assisted digestion parameters were as follows: max power 600 W, ramp time 10 min, pressure 350 psi, and temperature 130 °C for 10 min. Upon completion of the digestion process, vessels were allowed to cool at room temperature for 30 min. Digested materials were transferred to Eppendorf tubes and centrifuged at 10,000 rpm for three minutes to remove any undigested material. Digested materials (200 μL) were diluted in 9.8 mL of deionized water in 15 mL tubes and then analyzed by ICP-MS. To avoid contamination of samples, all PTFE material (Teflon vessels, pipettes, micro pipettes, tips and autosampler cups) were immersed in freshly prepared 15% v/v HNO_3_ for 2 h before analysis, then rinsed thoroughly with deionized water and dried in a dust free area before use. The blank digests and filtered residues went through a similar protocol of sample preparation and all samples were blank-corrected. In the case of filtered residue extracts (filtering), the concentration of most elements were below detection limits and in exceptional cases very close to the detection limits, indicating that metallic contents staying behind in undigested material had negligible significance.

### ICP-MS analysis

All samples, standards and reference materials, were analyzed in triplicate. An instrument auto-tune was performed using instrument verification standard (Tune-A) at 10 ppb in 2% nitric acid. A multi-element six point calibration standard curve was generated at 50, 100, 150, 200, 250 and 300 ppb. Samples were diluted in 2% nitric acid and aspirated by using the auto-sampler. The concentration was calculated from the dilution corrected values of the elements. Complete instrumental parameters are highlighted in (Table 1). Statistical evaluation of the results was carried out by Microsoft office Excel 2003. The proposed method was validated by undertaking a comparative study of the microwave-assisted digestion against certified reference material; Oriental tobacco leaves (CTA-OTL-1). Certified reference material was microwave-assisted digested and further analyzed by ICP-MS. Percentage relative standard deviation (% R.S.D.) was calculated by using recovered value of each metal from the proposed method and certified value. The percentage recovery of each metal was calculated as:

(1) % Recovery=100×value of proposed method/certified value

**Table 1:** Operating conditions for Plasma & ICP-MS

**Instrument**	**X7-Series ICP-MS System, Thermo Elemental Software, Version 131072**
Power	1403.92 W
Cool gas flow rate	13 L/min
Nebuilizer gas pressure	0.84 bar
Nebuilizer gas flow rate	0.82 L/min
Auxiliary gas flow rate	0.7 L/min
Condenser temperature	15 °C
Extract lens 1 voltage	3.8 V
Extract lens 2 voltage	−105.1 V
Extract lens 3 voltage	−195.29 V
Pole bias	1.53 V
Hexapole bias	6.47 V
Data acquisition	Using peri pump at 31 rpm
Main run setup	Peak jumping, Sweeps 100, Dwell time 10 (ms), Channels per mass 1, Channel spacing 0.02 AMU
No. of replicates	3
Sampler cone i.d.	1.1 mm
Skimmer cone i.d.	0.75 mm
External drift correction	Yes

### Statistical analysis

Multivariate statistical analysis can help to interpret data in a much easier and understandable manner. In the present study, simple XLSTAT (version 2011.4.04) was used for three multivariate techniques. There were 12 columns expressing elements and 55 rows indicating individual products. For a detailed statistical evaluation, principal component analysis was done on the whole data. It was done by diagonalizing the correlation matrix and all values were set at maximum unit variance, so the difficulty in analyzing different ranges of data points was avoided. The variability of the whole data was projected onto a scale, dividing variance into sub classes called principal components or factors. Moreover, Q-mode factor analysis after Varimax rotation was performed to check the variability among the tobacco products.

## Results and discussion

### Analytical figures of merit

The calibration curve for each metal and metalloid was constructed by using concentration range from the detection limit up to 50, 100, 150, 200, 250 and 300 ppb. The linearity, (r) value in all cases was 1 > r > 0.999. The limit of detection, LOD, was determined by aspiring ultra pure water as blank and signal intensities were recorded. A solution of 5 μg/L for each element was aspired and the signal intensities for all metals and metalloids were recorded. LOD was calculated by using equation:

(2) $$LOD=3.SD_{\mathrm{blank}}.Conc._{\mathrm{sample}}/I_{\mathrm{net}}$$

Where SD_blank_ is the standard deviation for the signal recorded on the blank for the corresponding element studied, Concn._sample_ is concentration in μg/L of the respective sample aspired, I_net_ = [I_sample_ – I_blank_], I_sample_& I_blank_ are the signal intensities recorded for the sample and blank, respectively. The LODs for all metals are summarized in (Table 2). Moreover, all samples above their LOD can be quantified, if their quantification limit would be approximately ten times the limit of detection.

**Table 2:** Linear regression data for the calibration curve of each element (n = 3)

**Element**	**Linearity, r**	**SD_blank_^a^**	**I_blank_**	**I_net_**	**LOD**
			**(CPS)^b^**	**(CPS)^b^**	**(ng/L)**
Al	0.999997	1.388	420199.36	26159.85	0.796
Cr	0.999969	0.023	8422.57	45971.71	0.008
Mn	0.999493	0.049	16561.75	63241.03	0.012
Fe	0.999989	0.417	602352.49	59517.32	0.100
Co	0.999830	0.005	661.02	55386.24	0.001
Ni	0.999907	0.038	4008.22	12804.31	0.045
Cu	0.999901	0.023	2908.8	15433.35	0.022
Zn	0.999973	0.058	844.04	9382.04	0.093
As	0.999986	0.069	−1311.36	8012.03	0.129
Se	0.999905	0.255	−42.93	730.7	5.235
Cd	0.999980	0.008	58.33	8784.3	0.014
Pb	0.999935	0.007	37.33	28190.14	0.004

^a^Standard deviation of twelve measurements; ^b^Counts per second.

### Recovery study

Recoveries of target elements were computed by comparison of microwave-assisted digestion data against certified reference material (CRM) values. Results showed that there was a good agreement between the recovered and certified values of the metal and metalloid contents in certified reference material. Percentage recovery of all elements was 96.1 < M < 108.5%, while percentage relative standard deviation (% R.S.D.) was < 5.8 in all cases. % Recovery of metal and metalloid content as evaluated by comparing the recovered and certified values is summarized in (Table 3).

**Table 3:** Validation of the micro-wave assisted digestion method against certified reference material (Oriental tobacco leaves, CTA-OTL-1), n = 6

**Element**	**Certified value ± S.D. (μg/g)**	**Recovered value ± S.D. (μg/g)**	**% R.S.D.**	**% Recovery**
^27^Al	252 ± 49	264.03 ± 5.14	3.29	104.77
^52^Cr	0.991^a^	1.07 ± 0.07	5.42	107.97
^55^Mn	136 ± 5	141.59 ± 3.2	2.85	104.11
^56^Fe	258^a^	248.11 ± 4.77	2.76	96.17
^59^Co	0.154^b^ ± 0.007	0.167^b^ ± 0.009	5.73	108.44
^60^Ni	1.49 ± 0.14	1.525 ± 0.076	1.64	102.35
^65^Cu	5.12 ± 0.2	5.48 ± 0.02	4.8	107.03
^66^Zn	43.6 ± 1.4	44.85 ± 0.05	1.99	102.87
^75^As	0.138^b^ ± 0.01	0.144^b^ ± 0.01	3	104.35
^82^Se	NA^c^	BDL^d^	NA^c^	NA^c^
^111^Cd	2.23 ± 0.12	2.386 ± 0.005	4.78	106.99
^208^Pb	0.972^b^ ± 0.147	0.965^b^ ± 0.028	0.51	99.28

^a^ Information value; ^b^ ng/g; ^c^ Not available; ^d^ Below detection limit.

### Quantification of metals and metalloids in various tobacco products

#### Tobacco smoked products

**Figure 1:**
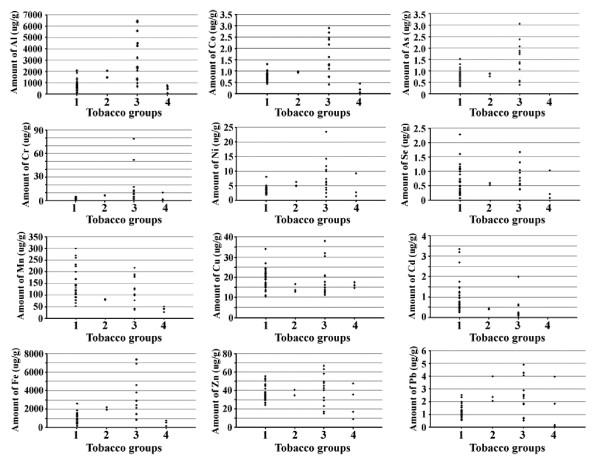
Graphical presentation of metals and metalloids contents in smoked 1, sniffing 2, dipping 3, and chewing 4 tobacco products.

Thirty-five samples of tobacco smoked products, including 26 Pakistani, 6 foreign brands of cigarettes, 2 brands of beedi and tobacco leaves (used for hookah smoking) were investigated for the quantification of metals and metalloids by the developed method. Iron and aluminum were the major elements among the all analyzed elements and were found in the range of 190–2600 and 150–2100 μg/g, respectively. Mn was in the range of 53–300 μg/g as the third most abundant element in all smoked tobacco products, while Pb, Cd, Se, Co, and As were present in < 3.4 μg/g. Fe and Al having similar a range in national and foreign smoked brands, but their quantity was high in beedi samples. Foreign cigarette brands contain more Mn and Zn contents as compared to other smoked tobacco products. There was no significant difference in Ni contents of different types of smoked tobacco products. As and Cd have almost similar levels in different smoked tobacco products. Se was found in trace quantities in all tobacco smoked products and it was below the detection limit in some cases. Pb contents have similar trends in Pakistani cigarette brands, but in the case of foreign brands there were variations in its concentration. Metal and metalloid contents in all tobacco products are summarized in (Table 4) and graphically presented in Figure 1.

**Table 4:** Metals and metalloids content in various tobacco products

**Code**	**Amount of element μg/g ± S.D.**											
	**Al**	**Cr**	**Mn**	**Fe**	**Co**	**Ni**	**Cu**	**Zn**	**As**	**Se**	**Cd**	**Pb**
Smoked tobacco products												
T1	820 ± 5.00	3.10 ± 0.02	110 ± 0.90	1200 ± 8.46	0.83 ± 0.01	3.92 ± 0.10	24.5 ± 0.11	38.6 ± 0.15	0.65 ± 0.02	BDL	0.68 ± 0.01	1.42 ± 0.01
T2	360 ± 0.50	1.49 ± 0.05	145 ± 0.30	460 ± 0.41	0.51 ± 0.02	2.36 ± 0.06	23.0 ± 0.06	34.0 ± 0.20	0.40 ± 0.02	0.29 ± 0.01	0.80 ± 0.04	0.65 ± 0.01
T3	1000 ± 1.47	3.53 ± 0.05	120 ± 0.15	1500 ± 0.75	1.00 ± 0.01	3.94 ± 0.11	34.0 ± 0.20	45.0 ± 0.25	0.77 ± 0.12	BDL	0.61 ± 0.02	1.89 ± 0.01
T4	603 ± 1.33	3.21 ± 0.06	90.4 ± 0.10	905 ± 3.55	0.64 ± 0.03	4.19 ± 0.20	18.8 ± 0.20	26.5 ± 0.23	0.91 ± 0.09	1.25 ± 0.06	0.54 ± 0.03	1.09 ± 0.05
T5	635 ± 14.30	2.76 ± 0.05	199.6 ± 0.4	872 ± 18.20	0.78 ± 0.01	3.94 ± 0.26	22.4 ± 0.36	34.4 ± 1.19	0.63 ± 0.02	BDL	1.10 ± 0.05	1.59 ± 0.05
T6	870 ± 5.50	3.52 ± 0.02	131.1 ± 0.3	1300 ± 5.70	0.90 ± 0.01	4.26 ± 0.05	23.8 ± 0.15	33.9 ± 0.10	0.87 ± 0.04	0.64 ± 0.21	0.66 ± 0.03	1.29 ± 0.01
T7	588 ± 2.78	2.45 ± 0.05	170 ± 0.92	790 ± 1.21	0.68 ± 0.01	2.24 ± 0.15	16.43 ± 0.1	35.4 ± 0.17	0.39 ± 0.05	BDL	1.00 ± 0.02	0.92 ± 0.01
T8	1150 ± 16.00	3.44 ± 0.05	105 ± 1.47	1400 ± 17.7	0.81 ± 0.01	3.61 ± 0.07	24.2 ± 0.30	36.7 ± 3.21	0.87 ± 0.07	0.66 ± 0.14	0.52 ± 0.01	1.18 ± 0.05
T9	517 ± 6.39	1.92 ± 0.09	142 ± 1.59	724 ± 5.09	0.64 ± 0.02	2.33 ± 0.20	15.75 ± 0.1	32.9 ± 0.10	0.89 ± 0.11	1.60 ± 0.14	1.22 ± 0.10	0.78 ± 0.01
T10	803 ± 8.82	3.17 ± 0.05	103 ± 0.75	1200 ± 9.53	0.85 ± 0.01	4.15 ± 0.15	26.9 ± 0.15	38.4 ± 0.43	0.81 ± 0.09	0.24 ± 0.09	0.63 ± 0.05	1.37 ± 0.05
T11	507 ± 15.60	3.07 ± 0.05	76.8 ± 0.26	840 ± 1.20	0.58 ± 0.01	2.33 ± 0.10	21.9 ± 0.05	29.5 ± 0.05	0.57 ± 0.09	0.17 ± 0.18	0.36 ± 0.03	1.01 ± 0.01
T12	894.8 ± 30.40	2.05 ± 0.14	66.12 ± 1.9	886.8 ± 28	0.44 ± 0.02	2.18 ± 0.21	20.7 ± 0.75	32.44 ± 1.4	1.08 ± 0.21	0.50 ± 0.12	0.3 ± 0.025	0.87 ± 0.05
T13	488 ± 3.70	3.00 ± 0.05	232 ± 0.20	623 ± 1.34	0.84 ± 0.02	2.52 ± 0.10	21.5 ± 0.05	52.5 ± 0.20	0.65 ± 0.12	0.62 ± 0.15	1.23 ± 0.03	0.61 ± 0.01
T14	520 ± 4.51	3.06 ± 0.10	78.9 ± 0.66	832 ± 8.17	0.60 ± 0.01	2.27 ± 0.15	16.5 ± 0.15	24.5 ± 0.15	0.51 ± 0.07	0.062 ± 0.1	0.43 ± 0.01	0.98 ± 0.02
T15	399 ± 0.60	2.08 ± 0.10	167 ± 0.10	541 ± 1.73	0.54 ± 0.01	2.27 ± 0.17	15.2 ± 0.11	35.8 ± 0.25	0.61 ± 0.10	0.73 ± 0.07	1.76 ± 0.03	1.08 ± 0.10
T16	1310 ± 12.20	4.25 ± 0.05	143 ± 1.65	1870 ± 21.8	1.30 ± 0.02	4.67 ± 0.05	21.9 ± 0.20	37.9 ± 0.25	1.00 ± 0.10	BDL	0.54 ± 0.03	1.72 ± 0.01
T17	1420 ± 5.60	4.19 ± 0.05	122 ± 0.45	1480 ± 7.70	0.85 ± 0.01	3.30 ± 0.10	22.0 ± 0.10	37.5 ± 0.10	0.81 ± 0.07	0.21 ± 0.04	0.47 ± 0.02	1.29 ± 0.02
T18	886 ± 9.12	2.93 ± 0.02	109.7 ± 1.0	935 ± 10.80	0.68 ± 0.01	3.06 ± 0.10	20.5 ± 0.06	29.8 ± 0.10	0.50 ± 0.01	BDL	0.73 ± 0.03	1.22 ± 0.01
T19	640 ± 1.00	3.50 ± 0.10	104 ± 0.45	1030 ± 2.12	0.74 ± 0.02	2.61 ± 0.26	19.0 ± 0.17	29.7 ± 0.55	0.86 ± 0.03	0.47 ± 0.04	0.45 ± 0.04	1.06 ± 0.05
T20	566 ± 1.90	2.93 ± 0.05	89.3 ± 0.15	844 ± 2.02	0.63 ± 0.01	2.25 ± 0.23	19.2 ± 0.20	27.0 ± 0.23	0.51 ± 0.04	BDL	0.50 ± 0.03	0.99 ± 0.01
T21	865 ± 7.75	3.67 ± 0.05	103 ± 1.21	1300 ± 12.5	0.84 ± 0.01	2.69 ± 0.06	19.2 ± 0.10	30.2 ± 0.25	0.94 ± 0.10	1.06 ± 0.21	0.55 ± 0.01	1.29 ± 0.01
T22	642 ± 5.43	2.79 ± 0.06	93.0 ± 0.72	887 ± 8.63	0.72 ± 0.01	2.72 ± 0.10	20.3 ± 0.10	27.0 ± 0.26	0.69 ± 0.12	0.64 ± 0.07	0.49 ± 0.03	1.00 ± 0.01
T23	449 ± 7.09	2.17 ± 0.05	118 ± 2.04	647 ± 10.03	0.70 ± 0.01	2.40 ± 0.05	20.4 ± 0.30	32.0 ± 0.43	0.46 ± 0.06	0.27 ± 0.05	0.74 ± 0.03	1.18 ± 0.05
T24	633 ± 6.59	2.90 ± 0.06	145 ± 1.02	967 ± 7.55	0.81 ± 0.01	3.66 ± 0.06	19.9 ± 0.15	35.3 ± 0.10	0.96 ± 0.10	1.10 ± 0.07	0.74 ± 0.03	1.19 ± 0.10
T25	669 ± 3.41	2.62 ± 0.05	91.96 ± 0.4	907 ± 0.85	0.68 ± 0.01	2.39 ± 0.10	19.0 ± 0.15	27.0 ± 0.20	0.60 ± 0.07	BDL	0.44 ± 0.01	1.06 ± 0.03
T26	382 ± 0.62	2.19 ± 0.10	170 ± 0.10	570 ± 1.96	0.86 ± 0.01	5.09 ± 0.09	21.5 ± 0.37	47.0 ± 0.70	0.60 ± 0.03	0.34 ± 0.04	1.27 ± 0.04	0.57 ± 0.01
I1	710 ± 2.52	1.84 ± 0.07	223 ± 1.06	600 ± 1.05	0.93 ± 0.01	2.23 ± 0.05	14.9 ± 0.11	53.3 ± 0.23	0.33 ± 0.01	BDL	0.96 ± 0.03	0.64 ± 0.01
I2	150 ± 1.10	1.68 ± 0.05	260 ± 2.53	190 ± 1.36	0.65 ± 0.01	1.92 ± 0.07	10.5 ± 0.11	52.6 ± 0.20	1.16 ± 0.28	2.28 ± 0.28	1.49 ± 0.07	1.03 ± 0.02
I3	320 ± 1.94	2.36 ± 0.05	225 ± 1.93	460 ± 2.08	0.89 ± 0.01	3.76 ± 0.15	17.4 ± 0.11	50.4 ± 0.05	0.82 ± 0.13	0.99 ± 0.02	3.35 ± 0.05	2.52 ± 0.01
I4	620 ± 1.57	1.66 ± 0.05	270 ± 8.67	570 ± 3.55	0.90 ± 0.02	2.43 ± 0.05	16.0 ± 0.11	42.2 ± 0.15	0.72 ± 0.06	0.39 ± 0.02	2.70 ± 0.01	2.36 ± 0.05
I5	300 ± 3.81	2.03 ± 0.05	200 ± 2.90	530 ± 6.35	0.76 ± 0.01	8.07 ± 0.05	15.0 ± 0.30	32.2 ± 0.32	0.38 ± 0.02	BDL	1.46 ± 0.06	0.56 ± 0.01
I6	330 ± 4.73	1.55 ± 0.05	300 ± 13.60	390 ± 3.15	1.03 ± 0.01	2.58 ± 0.10	17.0 ± 0.15	55.6 ± 0.40	0.72 ± 0.27	1.10 ± 0.10	3.20 ± 0.05	1.55 ± 0.05
B1	360 ± 3.50	1.70 ± 0.03	53 ± 0.25	440 ± 3.36	0.52 ± 0.02	3.70 ± 0.08	11.0 ± 0.24	38.0 ± 0.11	1.30 ± 0.28	0.73 ± 0.18	0.27 ± 0.03	0.79 ± 0.01
B2	1900 ± 30.20	3.90 ± 0.10	89.9 ± 1.19	1600 ± 20.0	0.88 ± 0.03	3.60 ± 0.07	13.0 ± 0.05	31.0 ± 0.40	0.74 ± 0.09	0.89 ± 0.13	0.37 ± 0.01	1.32 ± 0.03
B3	2100 ± 5.66	5.37 ± 0.10	140 ± 0.34	2600 ± 7.69	1.31 ± 0.02	4.47 ± 0.10	13.7 ± 0.14	45.7 ± 0.72	1.53 ± 0.18	1.16 ± 0.09	0.50 ± 0.08	2.00 ± 0.01
Sniffing tobacco products												
N1	1496 ± 9.28	6.33 ± 0.05	83.6 ± 0.10	1940 ± 14.2	0.98 ± 0.01	4.87 ± 0.13	12.8 ± 0.05	34.9 ± 0.20	0.87 ± 0.10	0.52 ± 0.09	0.42 ± 0.02	2.38 ± 0.01
N2	2080 ± 15.00	6.86 ± 0.04	82.3 ± 0.51	2220 ± 8.57	0.93 ± 0.01	5.07 ± 0.09	16.6 ± 0.05	40.8 ± 0.15	0.90 ± 0.07	0.59 ± 0.01	0.46 ± 0.01	2.07 ± 0.02
N3	1500 ± 19.67	7.14 ± 0.13	79.2 ± 0.73	1980 ± 19.4	0.93 ± 0.01	6.32 ± 0.30	13.6 ± 0.23	34.9 ± 0.51	0.77 ± 0.18	BDL	0.42 ± 0.01	3.99 ± 0.05
Dipping tobacco products												
N4	1000 ± 4.81	2.89 ± 0.03	41.5 ± 0.15	908 ± 5.36	0.76 ± 0.03	2.52 ± 0.15	17.7 ± 0.15	17.3 ± 0.30	0.40 ± 0.17	0.37 ± 0.12	0.10 ± 0.02	0.53 ± 0.01
N5	670 ± 1.22	2.12 ± 0.01	36.6 ± 0.11	840 ± 1.36	0.41 ± 0.01	1.19 ± 0.05	37.9 ± 0.15	29.5 ± 0.05	0.58 ± 0.17	0.56 ± 0.03	0.19 ± 0.01	0.74 ± 0.01
N6	1400 ± 15.50	5.42 ± 0.05	42.8 ± 0.32	1440 ± 12.2	1.10 ± 0.07	3.60 ± 0.02	16.2 ± 0.35	15.4 ± 0.05	0.53 ± 0.19	0.38 ± 0.02	0.11 ± 0.01	0.69 ± 0.02
N7	6500 ± 40.07	12.8 ± 0.10	217 ± 1.03	7400 ± 21.3	2.47 ± 0.02	11.7 ± 0.05	31.9 ± 0.50	58.3 ± 0.25	2.39 ± 0.15	0.95 ± 0.21	0.25 ± 0.01	4.25 ± 0.05
N8	6400 ± 6.87	13.4 ± 0.10	183 ± 0.35	7340 ± 23.6	2.70 ± 0.05	10.5 ± 0.05	30.4 ± 0.20	63.2 ± 0.10	3.07 ± 0.22	1.66 ± 0.35	0.26 ± 0.02	4.90 ± 0.25
N9	4300 ± 13.34	52.0 ± 0.28	130 ± 0.69	4600 ± 31.0	2.17 ± 0.01	14.24 ± 0.1	13.8 ± 0.15	42.7 ± 0.05	1.39 ± 0.10	1.04 ± 0.15	0.15 ± 0.01	1.87 ± 0.02
N10	4500 ± 15.89	17.6 ± 0.15	178 ± 0.90	2130 ± 13.7	2.39 ± 0.05	9.98 ± 0.20	20.5 ± 0.20	49.8 ± 0.10	2.08 ± 0.05	1.67 ± 0.35	0.22 ± 0.03	4.27 ± 0.05
N11	3200 ± 15.10	8.17 ± 0.06	104 ± 0.20	3800 ± 11.5	1.62 ± 0.01	6.41 ± 0.10	14.9 ± 0.10	32.4 ± 0.25	1.74 ± 0.17	0.52 ± 0.04	0.14 ± 0.01	4.06 ± 0.05
N12	2420 ± 16.20	8.53 ± 0.01	77.5 ± 0.35	2440 ± 10.9	1.27 ± 0.02	6.09 ± 0.13	11.3 ± 0.05	23.4 ± 0.11	1.32 ± 0.12	1.31 ± 0.01	2.00 ± 0.01	2.44 ± 0.02
N13	5600 ± 30.05	78.8 ± 0.15	189.8 ± 1.2	6900 ± 56.3	2.90 ± 0.07	23.5 ± 0.26	20.9 ± 0.15	48.4 ± 0.25	1.89 ± 0.20	0.37 ± 0.02	0.21 ± 0.02	2.29 ± 0.01
IN1	2340 ± 23.20	11.4 ± 0.05	128.5 ± 1.0	2930 ± 22.1	1.30 ± 0.01	7.36 ± 0.06	12.6 ± 0.03	45.3 ± 0.43	1.33 ± 0.08	0.67 ± 0.24	0.65 ± 0.04	2.54 ± 0.04
IN2	1300 ± 12.80	4.80 ± 0.06	98.5 ± 0.75	1500 ± 11.8	0.74 ± 0.01	4.32 ± 0.24	12.2 ± 0.05	66.9 ± 0.32	1.08 ± 0.20	0.77 ± 0.43	0.61 ± 0.02	1.80 ± 0.03
IN3	2100 ± 16.14	9.06 ± 0.47	124 ± 0.70	2900 ± 19.1	1.25 ± 0.01	5.37 ± 0.05	13.1 ± 0.15	40.5 ± 0.30	1.81 ± 0.17	0.57 ± 0.03	0.62 ± 0.02	2.89 ± 0.05
Chewing tobacco products												
G1	111.27 ± 8.17	0.69 ± 0.07	27.37 ± 0.7	176.96 ± 5	BDL	1.50 ± 0.07	16.0 ± 0.55	9.15 ± 0.27	BDL	1.03 ± 0.11	BDL	0.08 ± 0.00
G2	736.6 ± 24.90	10.44 ± 0.3	40.0 ± 1.03	762.1 ± 20	0.45 ± 0.02	9.27 ± 0.51	17.5 ± 0.42	17.07 ± 0.6	BDL	BDL	BDL	0.18 ± 0.01
G3	454 ± 14.70	1.95 ± 0.10	41.87 ± 0.9	563.1 ± 13	0.19 ± 0.00	2.75 ± 0.17	16.0 ± 0.29	47.72 ± 1.1	BDL	0.21 ± 0.04	BDL	1.85 ± 0.07
G4	542.7 ± 20.70	1.12 ± 0.09	51.07 ± 1.3	580.2 ± 14	0.06 ± 0.01	1.46 ± 0.15	14.68 ± 0.4	35.75 ± 1.1	BDL	0.07 ± 0.01	BDL	3.96 ± 0.16

#### Sniffing tobacco brands

Three local samples of sniffing tobacco (sniffing niswar) brands were investigated for the quantification of metals and metalloids by the proposed method. Fe, Al and Mn were the most abundant elements and present in higher concentrations in comparison to tobacco smoked products. Mn was found in the range of 79.2-83.6 μg/g, and lower in concentration in comparison to tobacco smoked brands. In the sniffing tobacco products, Pb was found in the range of 2.07-3.99 μg/g, more than that of smoked tobacco products. Cd was the minor trace metal, while Se was below the detection limit in sample N3. The results are presented in Table 4 and Figure 1.

#### Dipping tobacco brands

Ten local and three foreign samples of dipping tobacco (Niswar) brands were analyzed. Fe, Al and Mn were the principal components also in dipping tobacco samples. Fe and Al were found in the ranges of 840–7400 and 670–6500 μg/g, respectively. Most of the dipping tobacco samples contained higher concentration of metal and metalloids than all other types of tobacco products, except Mn, Se and Cd. No significant difference of elemental contents was observed between local and imported dipping tobacco products except in two Pakistani products, N7 and N8 (Table 4) and Figure 1.

#### Chewing tobacco brands

Four brands of gutka (a chewing tobacco product), which is commonly consumed in Pakistan, were investigated. Generally, they contained metals in fewer quantities, except Cr, Ni, Cu, Zn and Se. Three major elements Fe, Al and Mn, showed similar trends, to that observed in other tobacco products. A gutka sample, G2 had Cr and Ni in concentrations of 10.44 and 9.27 μg/g, respectively, more than smoked and sniffing tobacco brands. Similarly, the amount of Cu (17.5 μg/g) in G2 was higher than the sniffing tobacco products. The amount of Zn (47.72 μg/g) in G3 was more than in the sniffing tobacco brands. Se (1.03 μg/g) in G1 was more than the sniffing tobacco products, while Co was below the detection limit in the same sample. As and Cd were below the detection limit in all chewing tobacco brands. Results are summarized in Table 4 and in Figure 1. Codes of all samples have been decoded in Additional file 1: Table S1.

**Figure 2:**
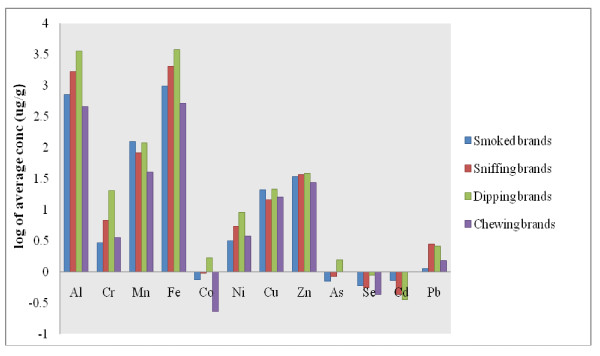
Graphical representation of average concentration of metals and metalloids in various tobacco groups.

A mean value of all analyzed samples from each category of tobacco products was calculated for various elements and a log-scale comparative graph was drawn Figure 2. The decreasing order of elemental concentration can be summarized as Fe > Al > Mn > Zn > Cu > Ni > Cr > Pb > Co > Cd > As > Se for smoked tobacco products, Fe > Al > Mn > Zn > Cu > Cr > Ni > Pb > Co > As > Se > Cd for sniffing tobacco products, Fe > Al > Mn > Zn > Cu > Cr > Ni > Pb > Co > As > Se > Cd for dipping tobacco products and Fe > Al > Mn > Zn > Cu > Ni > Cr > Pb > Se > Co > As > Cd for chewing tobacco products. Sniffing, dipping and chewing products are different from the smoked products due to their direct contact of tobacco into mucus membrane and metals can be absorbed through mucosal membrane. The recommended dietary intake of metals and metalloids [26-28] is highlighted in the Additional file 1: Table S2. The situation is really alarming as the single dose of these tobacco products is between 2–10 g, while the number of doses consumed per day mainly depends on the level of tobacco addiction of the user, this would be an additional amount of metals taken by the consumer excluding other environmental and nutritional sources.

#### Comparison with existing data

Many researchers have quantified the metal contents in smoked tobacco products, particularly in cigarettes [29]. We have compared our data with the reported literature of smoked tobacco products from Pakistan (Table 5). Limited literature is available about sniffing and chewing products and only from India. However, to the best of our knowledge, no report exists about metal contents in dipping tobacco products. In smoked products, our study showed that Mn, Cu and Zn were higher; Cd has comparable concentration, while Co and Pb were lower than the reported concentrations from Pakistan [21]. In the case of sniffing tobacco, most of the metallic contents are nearly comparable with the existing data [30], while Mn was found to be lower and Pb as reported by U.C. Mishra et al. [31] was reasonably high among all the reported studies. In case of chewing tobacco, our results showed a little lower concentration of Cr, Mn, Fe and Pb, when compared with existing literature [29-33]. Only one report exists about the quantification of the metalloid, Arsenic, in a chewing tobacco product, but As levels were below the detection limit in our case. The present work is the latest source of information with reference to metals and metalloids contents in tobacco products, especially smokeless tobacco formulations.

**Table 5:** Metal and metalloid concentration levels (μg/g) in reported literature

**Product**	**Al**	**Cr**	**Mn**	**Fe**	**Co**	**Ni**	**Cu**	**Zn**	**As**	**Se**	**Cd**	**Pb**	**Reference**
Smoked	-	-	45.03	-	3.344	-	7.889	14.34	-	-	0.501	14.39	21
	716	2.92	124.04	981.18	0.75	3.13	21.1	34.27	0.71	0.6	0.72	1.13	Present study
Sniffing	-	15.6	-	2972	-	9.13	16	37	-	-	0.48	3.1	21
	-	~3.8	~124.4	-	~0.47	~3.0	~28	~42	-	-	-	~0.78	30
	-	-	-	2090	-	2.1	21	43	-	-	-	8.0	31
	-	3.0	150	2323	0.4	-	-	46	-	-	-	-	32
	1692	6.78	81.7	2046.67	0.95	5.42	14.33	36.87	0.85	0.56	0.43	2.81	Present study
Dipping	3599	20.17	120.02	3779.8	1.68	8.97	21.55	38.04	1.54	0.89	0.36	2.6	Present study
Chewing	-	6.97	-	853	-	2.37	42	56	-	-	0.38	8.38	29
	-	~6.0	~174	~1100	~0.69	~1.5	~12	~28	~0.91	-	~0.8	~4.5	30
	-	-	-	1050	-	1.1	11	20	-	-	-	4.5	31
	-	6.3	127	1565	0.6	-	-	19	-	-	-	-	32
	-	6.6	129	1703	0.75	-	-	18.5	-	-	-	-	33
	461.14	3.55	40.08	520.59	0.23	3.75	16.05	27.42	BDL	0.44	BDL	1.52	Present study

*BDL* below detection limit.

## Statistical evaluations

### Correlation between variables

As the principal component analysis is based on Eigen values of correlation matrix, a detailed discussion on correlation matrix is useful in divergence or coherence of data. Data points, whose concentrations were below the detection limit, were replaced by half of detection limit values for the statistical evaluation. Values higher than 0.50 were considered to correlate the data points (Table 6). Positive values in the table show positive correlation among variables while negative values show negative correlation. Values closer to 0 indicate poor negative or positive correlation. However, values nearer to 1 show significant correlation. Neither pairs of elements showed significant negative correlation between them, however, Cu showed no appreciable correlation to any of the element analyzed. Hence, it was placed in a separate group. The remaining elements were easily grouped in two distinct classes after interpretation of the correlation matrix and are: Group 1 = Al, Fe, Co, Ni, As, Cr and Pb, Group 2 = Mn, Zn, Se and Cd, Group 3 = Cu. Grouping of the data is further elaborated through a multivariate analysis i.e. principal component analysis.

**Table 6:** Pearson Correlation matrix for 12 elements

**Variables**	**Al**	**Cr**	**Mn**	**Fe**	**Co**	**Ni**	**Cu**	**Zn**	**As**	**Se**	**Cd**	**Pb**
Al	**1**											
Cr	**0.673**	**1**										
Mn	0.159	0.121	**1**									
Fe	**0.962**	**0.683**	0.146	**1**								
Co	**0.929**	**0.712**	0.385	**0.893**	**1**							
Ni	**0.805**	**0.901**	0.188	**0.805**	**0.828**	**1**						
Cu	0.199	−0.003	0.034	0.227	0.184	0.064	**1**					
Zn	0.403	0.210	**0.651**	0.391	0.480	0.302	0.103	**1**				
As	**0.843**	0.450	0.295	**0.825**	**0.850**	**0.599**	0.148	**0.517**	**1**			
Se	0.315	0.100	0.272	0.235	0.317	0.127	−0.157	0.293	**0.546**	**1**		
Cd	−0.289	−0.209	**0.698**	−0.286	−0.077	−0.195	−0.171	0.291	−0.083	0.198	**1**	
Pb	**0.707**	0.270	0.174	**0.666**	**0.633**	0.438	0.045	0.469	**0.676**	0.229	−0.054	**1**

Values in bold show absolute values greater than 0.50.

#### Principal component analysis

**Figure 3:**
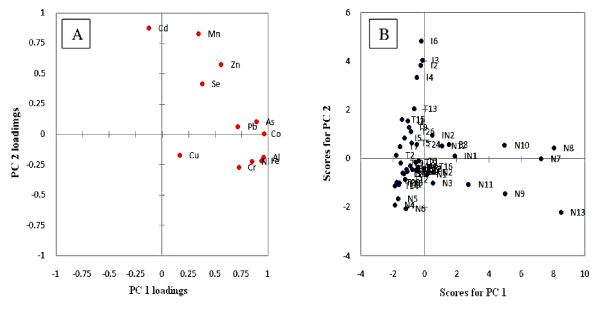
A = contribution of each element to the PC loadings and B = principal component scores for tobacco products.

Principal components having Eigen values greater than 1 were extracted for this study. This generated four independent components. The first component contributed as 49.1%, second 18.4%, third 9.3% and fourth 8.7% variability of the data. Total contribution from these four components is 85.7% of the total variation (Table 7. Analysis of Table 7 shows that elements of group 1 gave a major contribution to principal component 1. The second group of the correlation matrix significantly contributed in factor loadings of principal component 2. While a unique trend for Cu was again observed. It showed a significant appearance in principal component 3 and no other element appeared in that factor. This grouping was also clearly apparent in the diagram of loadings of components 1 and 2 (Figure 3A). This figure showed a total of 67.56% variance of the data, while, Figure 3B showed the score plot for the first two components.

**Table 7:** Principal component loadings

**Variable**	**PC1**	**PC2**	**PC3**	**PC4**
Al	**0.959**	−0.188	0.011	−0.091
Cr	**0.725**	−0.272	−0.234	0.509
Mn	0.344	**0.828**	0.157	0.302
Fe	**0.940**	−0.216	0.055	−0.035
Co	**0.964**	0.006	0.024	0.084
Ni	**0.846**	−0.227	−0.139	0.396
Cu	0.169	−0.172	**0.891**	−0.061
Zn	**0.559**	**0.577**	0.231	−0.006
As	**0.889**	0.103	−0.018	−0.322
Se	0.381	0.415	−0.409	−0.495
Cd	−0.123	**0.877**	−0.026	0.237
Pb	**0.716**	0.065	0.053	−0.352
Eigen value	5.896	2.211	1.122	1.056
Variability (%)	49.136	18.422	9.346	8.799
Cumulative Variance (%)	49.136	67.558	76.904	85.704

Values in bold show absolute values greater than 0.50.

#### Q-mode factor analysis

**Figure 4:**
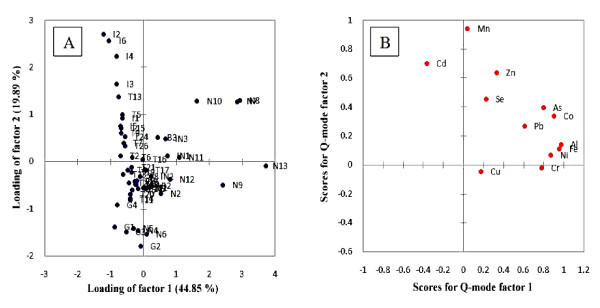
A = plot of Q-mode factor loadings and B = plot of Q-mode factor scores.

The 55 different tobacco products were subjected to Q-mode factor analysis. This analysis generated many factors, but among them, only two were produced after Varimax rotation. These two factors accounted for 64.7% of total variability (Table 8). The first factor with 44.8% variability was considered to be a major factor. This factor may depend on the processing procedure. As a majority of the smoked tobacco products (either local or international) have low loadings in factor 1 and all the smokeless products have high loadings in factor 1 it can be concluded that smokeless tobacco products undergo many processing steps and thus they have more chance of elemental contamination. The smoked tobacco products undergo fewer processing steps, hence majority of them gave high loadings in factor 2. Figure 4A and B showed the loadings and scores plot of factors 1 and 2 for the Q-mode factor analysis, respectively.

**Table 8:** Factor loadings of Q-mode factor analysis after Varimax rotation

**Sample Code**	**D1**	**D2**
T1	−0.243	−0.485
T2	**−0.706**	**0.117**
T3	−0.196	−0.398
T4	**−0.473**	**−0.186**
T5	**−0.636**	**1.003**
T6	**−0.349**	**0.086**
T7	**−0.593**	**0.397**
T8	−0.099	−0.314
T9	**−0.673**	**0.608**
T10	−0.227	−0.474
T11	−0.404	−0.817
T12	−0.001	−0.560
T13	**−0.763**	**1.376**
T14	−0.407	−0.786
T15	**−0.678**	**0.714**
T16	**−0.020**	**0.044**
T17	0.067	−0.173
T18	−0.265	−0.426
T19	**−0.371**	**−0.220**
T20	−0.395	−0.688
T21	**−0.361**	**−0.122**
T22	−0.447	−0.447
T23	**−0.622**	**−0.265**
T24	**−0.566**	**0.525**
T25	−0.334	−0.604
T26	**−0.566**	**0.333**
I1	**−0.640**	**0.928**
I2	**−1.212**	**2.712**
I3	**−0.814**	**1.646**
I4	**−0.829**	**2.243**
I5	**−0.697**	**0.756**
I6	**−1.050**	**2.562**
B1	−0.159	−0.571
B2	0.242	−0.506
B3	**0.419**	**0.520**
N1	0.115	−0.562
N2	0.524	−0.673
N3	0.069	−0.574
N4	−0.166	−1.469
N5	−0.302	−1.410
N6	0.094	−1.535
N7	2.844	1.279
N8	2.920	1.304
N9	2.404	−0.501
N10	1.626	1.280
N11	1.073	0.088
N12	0.796	−0.375
N13	3.718	−0.089
IN1	0.725	0.129
IN2	0.230	−0.312
IN3	0.676	0.488
G1	−0.871	−1.385
G2	−0.085	−1.791
G3	−0.531	−1.496
G4	−0.790	−0.920
Eigenvalue	5.780	2.000
Variability (%)	44.854	19.888
Cumulative Variance (%)	44.854	64.742

Values in bold show samples with low loadings in Factor 1 but high loadings in Factor 2.

## Conclusions

This study focused on ICP-MS based quantitative estimation of metals and metalloids in various tobacco products. The method was validated by analyzing certified reference material. Good agreement between recovered and certified values showed effective recovery of the metals after microwave digestion and subsequent accurate detection. The limits of detection for all twelve elements ranged between 0.001-5.23 ng/L, which were much better when compared with the studies reported in literature. The average observed concentration ranges (μg/g) of metals in all types of tobacco products were Al (111.27-6500), Cr (0.69-78.8), Mn (27.37-300), Fe (176.96-7400), Co (0.06-2.9), Ni (1.19-23.5), Cu (10.5-37.9), Zn (9.15-66.9), As (0.33-3.07), Se (0.062-2.28), Cd (0.093-3.35) and Pb (0.08-4.9). The present study provides reliable data about the metals distribution in some commonly consumed tobacco products. Hence, this study would be helpful for toxicologists and environmental chemists to evaluate the health effects of tobacco products and their contribution towards overdosing of the metals in tobacco users.

## Competing interests

The authors declare that they have no competing interests.

## Authors’ contributions

SGM: Participated in the experimental designing and method optimization. MS: Participated in bench work and played a role in paper writing. AJS: Involved in statistical evaluations. MNH: Involved in the useful discussion and participated in paper writing. AA: Involved in the useful discussion and participated in performing experiment. All authors read and approved the final manuscript.

## Supplementary Material

Additional file 1 **Table S1 Decoding of sample codes.**Table S2. Recommended dietary intake of metals and metalloids. Click here for file
