# Supplementary material for: Quantitative analysis of some important metals and metalloids in tobacco products by inductively coupled plasma-mass spectrometry (ICP-MS)
Source: Chem Cent J. 2012 Jun 18;6:56. doi: 10.1186/1752-153X-6-56 (PMC3443060; doi:10.1186/1752-153X-6-56)
Supplement: Additional file 1 — Table S1 Decoding of sample codes.Table S2. Recommended dietary intake of metals and metalloids. [file 1752-153X-6-56-S1.doc]

**Supplementary material**

**Table 1 Decoding of sample codes.**

| Code | Brand name | Code | Brand name |
| --- | --- | --- | --- |
| T1 | Master | I1 | Hi-Light |
| T2 | Benson & Hedges | I2 | Pine Menthol Lights |
| T3 | Gold Street Classic | I3 | Business Club |
| T4 | Princeton | I4 | Fisher |
| T5 | Park Lane Special | I5 | Dunhill |
| T6 | Wonder | I6 | Pine Lights |
| T7 | L & M Red | B1 | S-Beedi |
| T8 | Melburn | B2 | 25-Beedi |
| T9 | L & M Blue | B3 | Tobacco Leaves |
| T10 | Boss | N1 | Red Niswar |
| T11 | Hero | N2 | Brown Niswar |
| T12 | Gold Street | N3 | Black Niswar |
| T13 | Marlboro Lights | N4 | Manzor Kashmeeri Niswar |
| T14 | Wills Kings | N5 | Daood Kashmeeri Niswar |
| T15 | Dunhill | N6 | Green Niswar |
| T16 | Power | N7 | Black Simple Niswar |
| T17 | Royals Filter | N8 | Black Mesaly Wala Niswar |
| T18 | Capstan | N9 | Allaichi Wala Niswar |
| T19 | K2 | N10 | Chinar Gul Niswar |
| T20 | Gold Flak | N11 | Tank Marka Niswar |
| T21 | Embassy Filter | N12 | Gold Leaf Special Niswar |
| T22 | Diplomat | N13 | Mix Niswar |
| T23 | Gold Leaf | IN1 | Tara Filter Khaini |
| T24 | Red & White | IN2 | Hot Filter Khaini |
| T25 | Morven Gold | IN3 | Mahak Chaini Khaini |
| T26 | Marlboro | G3 | Rajguru Gutka |
| G1 | Doctor Gutka | G4 | Golden Gutka |
| G2 | J.M Gutka |  |  |

Codes incorporated with “I” represent international brands.

**Table 2** Recommended dietary intake of metals and metalloids.

| Metal | Tolerable daily intake | Unit |
| --- | --- | --- |
| Al | nf | nf |
| Cr | 50-200^b^ | µg/day |
| Mn | 2-5^a^ | mg/day |
| Fe | 10-15^a^ | mg/day |
| Co | nf | nf |
| Ni | 300^a^ | µg/day |
| Cu | 1.5-3^a^ | mg/day |
| Zn | 10-15^a^ | mg/day |
| As | 0.015^b,d^ | mg/week |
| Se | 55^c^ | µg/day |
| Cd | 60^a^ | µg/day |
| Pb | 214^a^ | µg/day |

nf = not found; a = [26]; b = [27];

c = [28]; d = mg/kg body weight.
